# Three counting methods agree on cell and neuron number in chimpanzee primary visual cortex

**DOI:** 10.3389/fnana.2014.00036

**Published:** 2014-05-16

**Authors:** Daniel J. Miller, Pooja Balaram, Nicole A. Young, Jon H. Kaas

**Affiliations:** Department of Psychology, Vanderbilt UniversityNashville, TN, USA

**Keywords:** stereology, isotropic fractionator, flow cytometry, cell density, neuron number

## Abstract

Determining the cellular composition of specific brain regions is crucial to our understanding of the function of neurobiological systems. It is therefore useful to identify the extent to which different methods agree when estimating the same properties of brain circuitry. In this study, we estimated the number of neuronal and non-neuronal cells in the primary visual cortex (area 17 or V1) of both hemispheres from a single chimpanzee. Specifically, we processed samples distributed across V1 of the right hemisphere after cortex was flattened into a sheet using two variations of the isotropic fractionator cell and neuron counting method. We processed the left hemisphere as serial brain slices for stereological investigation. The goal of this study was to evaluate the agreement between these methods in the most direct manner possible by comparing estimates of cell density across one brain region of interest in a single individual. In our hands, these methods produced similar estimates of the total cellular population (approximately 1 billion) as well as the number of neurons (approximately 675 million) in chimpanzee V1, providing evidence that both techniques estimate the same parameters of interest. In addition, our results indicate the strengths of each distinct tissue preparation procedure, highlighting the importance of attention to anatomical detail. In summary, we found that the isotropic fractionator and the stereological optical fractionator produced concordant estimates of the cellular composition of V1, and that this result supports the conclusion that chimpanzees conform to the primate pattern of exceptionally high packing density in V1. Ultimately, our data suggest that investigators can optimize their experimental approach by using any of these counting methods to obtain reliable cell and neuron counts.

## Introduction

Determining the cellular composition of neurobiological tissue is critical to our understanding of the structure and function of the brain. Differences in the organization of neurobiological tissue, whether it is the arrangement of cells or the relative amount of an organism's nervous system dedicated to a particular sensory modality, are important components of the neurobiological circuitry underlying behavior (Sur et al., 1980; Catania et al., 1993; Krubitzer et al., 1995).

Scientists have employed a variety of techniques with varying degrees of success to more fully characterize the microstructure and circuitry of specific brain areas and systems. An early example of such a procedure is “direct enumeration,” which consists of homogenizing brain tissue and determining the density of cell nuclei in re-suspended fluid samples (Nurnberger and Gordon, 1957; Zamenhof, 1976). A major critique of this approach was concern over the potential loss of cells or nuclei during the mechanical dissociation of tissue (Clarke and Oppenheim, 1995). Alternative procedures were developed to analyze specific parameters of sectioned tissue samples, and since the 1960's these stereological techniques have become the gold-standard for quantitative investigations of biological tissues (Glaser and Glaser, 2000). However, stereological methods have also been critiqued for inattention to tissue processing deformations that may lead to inaccurate estimates (von Bartheld, 2002). Traditionally, for example, guard zones were used to avoid tissue containing “lost caps” that resulted from cells being destroyed by direct contact with the knife blade (Baryshnikova et al., 2006). However, recent work suggests the absence of “lost caps” in fixed tissues cut on a freezing microtome, but reports that the distribution of particles along the z-axis (from one cut edge to the other) is non-uniform, concluding that the traditional use of guard zones may introduce bias into the estimate for these tissues (Carlo and Stevens, 2011). Indeed, tissue deformations that introduce bias into stereological investigations have been reported for a number of tissue processing protocols and constitute a major cause for concern (von Bartheld, 2012). Thus, the extent to which these distinct methodologies produce accurate estimates of specific biological parameters of interest remains controversial (Bahney and von Bartheld, 2014). Accordingly, whether these methods produce comparable results has also been of interest for several decades as agreement between methods provides support for the conclusion that we are, in fact, producing reasonable estimates of a specific biological parameter of interest, and forms the motivation for our work. An additional challenge to this type of work is the ability to accurately identify the region of interest for quantitative analysis. Immunohistochemical preparations have enhanced our ability to identify discrete brain areas by providing information on an increased number of the molecular properties of the brain (Sherwood et al., 2007a,b; Raghanti et al., 2008; Takahata et al., 2009; Balaram et al., 2013). Indeed, stereological analysis has seen widespread success in part because of the ability to employ immunohistochemical protocols to tag neurons (Mullen et al., 1992) as well as histological procedures to visualize cells, including the Nissl stain (Miller et al., 2012), and neurotransmitter biomarkers, such as acetylcholinesterase (Miller et al., 2013), in adjacent brain slices. Not only does this increased number of staining procedures give researchers more to investigate with the techniques at their disposal, it also provides an enriched view of the neurobiology under investigation and facilitates the rapid and reliable identification of brain areas. Importantly, recent years have witnessed the application of some of these newer technologies to older procedures. For example, the isotropic fractionator (Herculano-Houzel and Lent, 2005) is the product of combining “direct enumeration” with immunohistochemistry to investigate, for example, the number of neuronal and non-neuronal cells in a tissue sample. In addition, results from manual counts using the isotropic fractionator method (Herculano-Houzel and Lent, 2005) have been replicated using modern flow cytometry to more rapidly quantify the cellular composition of neurobiological samples, termed the flow fractionator (Collins et al., 2010; Young et al., 2012). A principal reason these procedures have seen widespread use in recent years is that each has specific advantages. For instance, while stereological analysis of brain slices provides a wealth of anatomical detail, the preparation of histological sections is time-consuming, and adequately sampling brain tissue can be challenging. Conversely, the isotropic fractionator can more rapidly produce reliable estimates of some of the same parameters, such as the cellular composition of a brain sample, via homogenization of samples (Herculano-Houzel and Lent, 2005). Recently, the use of flow cytometry to automatically count samples prepared using the isotropic fractionator was demonstrated to further decrease the amount of time necessary to estimate cellular populations, and to provide results less variable than those obtained from hand counts using a microscope, although with an increased equipment cost (Collins et al., 2010; Young et al., 2012). Furthermore, although stereological studies can employ a range of histological markers to identify individual regions of interest, the isotropic fractionator and flow fractionator can each be used for samples taken from cortex that has been separated from the white matter and flattened into a sheet, in which specific brain areas, such as the primary visual cortex (V1), are readily identifiable during dissection. Despite the theoretical ability of these procedures to estimate the same parameters, no study has to-date directly compared results from the use of the isotropic fractionator, the flow fractionator and the stereological optical fractionator in a single brain region.

The present study is therefore the first to investigate the similarity of estimates obtained using the isotropic fractionator, the flow fractionator and the stereological optical fractionator from a specific region of the brain within a single individual. We determined the total number of cells by staining free-floating suspensions of nuclei for DNA with 4',6-diamidino-2-phenylindole (DAPI), and the percentage of neuronal cells by staining suspensions of nuclei with both DAPI and an antibody to the neuronal nuclei marker NeuN (Mullen et al., 1992) in samples from flattened V1 of the right hemisphere using the isotropic fractionator (Herculano-Houzel and Lent, 2005; Collins et al., 2010; Young et al., 2012). We then determined the total number of cells and the percentage of neuronal cells in the same samples with the flow fractionator (Collins et al., 2010; Young et al., 2012). Finally, we recorded the location of cells along the z-axis to determine the presence and extent of possible non-uniform tissue shrinkage that could bias our stereological estimates, and calculated the total number of cells and neurons using the stereological optical fractionator (Schmitz and Hof, 2005) in adjacent series of brain slices from the left hemisphere stained for Nissl substance or NeuN, respectively. Determining the agreement in results between these methods is important because each of these methods are in current use and the reliability of our conclusions and inferences based upon such data will depend upon how they compare. In addition, if estimates from these methods agree, this will provide investigators with a greater degree of experimental flexibility and increase the data to which they have access when interpreting their results. In short, determining the transparency of results from these techniques facilitates the integration of a larger set of data in the development of formal relationships between the cellular composition and behavioral function of brain regions across a broad range of taxa and in both clinical and basic scientific applications.

## Methods

### Sample preparation

Both hemispheres of the brain from a single, non-neuropathological adult female chimpanzee (*Pan troglodytes*; 53 years) were obtained from the Texas Biomedical Research Institute's Southwest National Primate Research Center. The brain was collected after post-mortem flushing with 0.1% phosphate buffered saline (PBS), and was shipped overnight in 0.1% PBS. Upon arrival, the cerebral cortex was separated from the subcortical structures, bisected, and then cleaned of vasculature and pia. In the right hemisphere, the sulci were opened in order to flatten the cortical sheet, which was then fixed in 2% PFA in PBS for 72 h at 4°C. The left hemisphere was cut into blocks and fixed in 4% PFA in PBS for 24 h at 4°C, followed by immersion in 30% sucrose overnight prior to sectioning at 50 μm on a sledge microtome (American Optical, model #860, clearance angle = 19°) while the tissue was held below 0°C.

Details of the tissue processing methods employed in flattening cortex and preparing samples for the isotropic fractionator protocol appear in prior publications (Herculano-Houzel and Lent, 2005; Collins et al., 2010; Young et al., 2012). Briefly, the flattened (right) cortical hemisphere was photographed and placed onto a light box. Primary visual cortex (V1 or Brodmann's area 17) in flattened cortex is easily identifiable when backlit due to its dense myelination and caudal position. Identifiable cortical areas (V1) were drawn on the printed image of the flattened cortex and V1 was separated from the rest of cortex and subdivided into pieces using a scalpel. Each piece was photographed, weighed, and documented as a single sample. Photographs were taken in order to measure the surface area of each piece using ImageJ, which were combined with the average cortical thickness of V1 based upon measurements (mean depth = 1.8 mm, *n* = 42) in serial sections from the left hemisphere to determine the volume of V1. Each sample from the flattened cortical tissue (*n* = 61) was homogenized using a glass Tenbroeck tissue grinder (Fisher Scientific) in a dissociation solution of sodium citrate, triton-X 100 and distilled water. The total suspension volumes were determined based upon sample weight, and ranged between 3 and 5 ml. The resulting solution contains free-floating nuclei, which were stained with DAPI, which indiscriminately stains cellular nuclei, and an antibody to Neuronal Nuclei (NeuN, MAB377, Millipore), which stains most neuronal nuclei (Mullen et al., 1992). The isotropic fractionator and flow fractionator procedures described below permit the estimation of total cell number as well as the percentage of neurons in the sample, assuming that each nucleus corresponds to one cell.

### Isotropic fractionator

**Figure 1:**
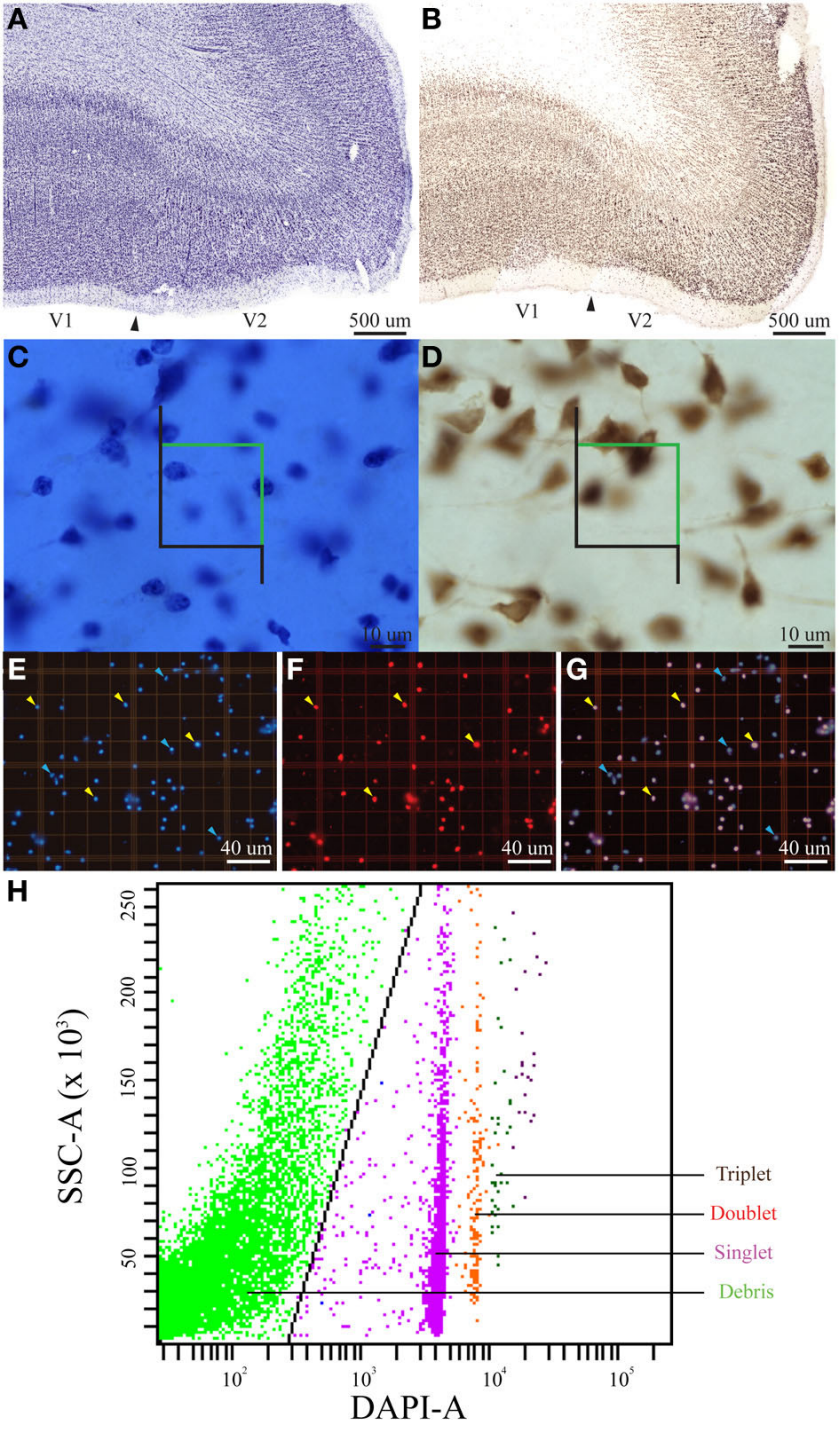
**Summary figure of the optical fractionator, isotropic fractionator, and flow fractionator**. Low magnification (~1×, scale bar = 500 μm) images of coronal brain slices at the V1/V2 cortical boundary (indicated by the arrowhead) stained for Nissl **(A)** and an antibody to NeuN **(B)** showing the characteristic laminar profile of V1 that was used to guide stereological measurements. High magnification images (100×, scale bar = 10 μm) of the outer stripe of Gennari in V1 in coronal brain slices stained for Nissl **(C)** and an antibody to NeuN **(D)**, with the stereological probe illustrating lines of inclusion (green) and exclusion (black). High magnification (20×, smallest individual boxes are 50 by 50 μm, scale bar = 40 μm) images of flattened cortical samples processed using the isotropic fractionator and quantified using the Neubauer chamber **(E,F,G)**. **(E)** Shows DAPI-stained nuclei, **(F)** shows NeuN-stained nuclei and **(G)** is a merged image of **(F,G)**. Yellow arrowheads indicate nuclei double-positive for fluorescent staining of DAPI and NeuN, indicating neuronal nuclei, and blue arrowheads indicate nuclei that stained for DAPI, but did not stain for NeuN, indicating non-neuronal nuclei **(E,F,G)**. **(H)** Shows an example of our data generated using the flow fractionator and presented on a SSC-A (side scatter area) vs. DAPI-A (DAPI-fluorescent area) scatterplot. A polygon gate was used to select the nuclei based on DAPI expression. This analysis profile allowed us to quantify the concentration of individual nuclei (singlet, teal), clusters of two nuclei (doublet, red) and clusters of three nuclei (triplet, black) in a known volume of sample. The green dots on the left side of the plot indicate debris excluded by the polygon gate (black diagonal line).

Nuclei in samples from the flattened (right) V1 and processed using the isotropic fractionator were stained with DAPI and an antibody to NeuN and quantified by hand in a Neubauer counting chamber under a fluorescent microscope (Herculano-Houzel and Lent, 2005). Specifically, free-floating, DAPI-stained, or DAPI+/NeuN+ double-stained nuclei from the main suspension samples were counted to estimate total cell number or percentage of neurons, respectively (Figure 1, Table 1). Initial manual counts of DAPI-stained or DAPI+/NeuN+ double-stained nuclei were performed by a technician blind to the flow cytometry data, and control samples revealed excellent agreement between the initial and final manual counts performed by DJM on DAPI-stained nuclei (nonparametric *T*-test, *P* = 0.967, *n* = 17), and between manual and flow counts of DAPI+/NeuN+ double-stained nuclei (nonparametric *T*-test, *P* = 0.123, *n* = 13). All suspensions were vigorously mixed prior to chamber loading and counting, and each of the 61 samples from right V1 was processed using the isotropic fractionator method.

**Table 1:** **Adult chimpanzee visual cortex total cell and neuron estimates based on three quantification techniques**.

**Tissue**	**Quantification**	**Parameter**	**Sample**	**Estimate**
Flat	Neubauer chamber	All cells	*n* = 61	998,480,148
Flat	Flow cytometry	All cells	*n* = 61	1,015,656,849
Slice	Optical fractionator	All cells	*n* = 324	961,086,450
Flat	Neubauer chamber	Neurons	*n* = 61	651,739,214
Flat	Flow cytometry	Neurons	*n* = 61	664,726,981
Slice	Optical fractionator	Neurons	*n* = 316	695,478,260
Flat	Neubauer chamber	Percent neurons	*n* = 13	62.0%
Flat	Flow cytometry	Percent neurons	*n* = 61	65.1%
Slice	Optical fractionator	Percent neurons	*n* = 10	72.4%
Flat	Image J	Volume (mm^3^)	*n* = 61	5275
Slice	Cavalieri (Nissl)	Volume (mm^3^)	*n* = 10	4819
Slice	Cavalieri (NeuN)	Volume (mm^3^)	*n* = 10	4778
Flat	Neubauer chamber	Cell density	*n* = 61	192,184
Flat	Flow cytometry	Cell density	*n* = 61	195,551
Slice	Optical fractionator	Cell density	*n* = 10	197,331
Flat	Neubauer chamber	Neuron density	*n* = 61	125,641
Flat	Flow cytometry	Neuron density	*n* = 61	128,005
Slice	Optical fractionator	Neuron density	*n* = 10	146,008

Summary table showing the parameter estimates obtained from flattened cortical samples (Flat) and brain slices (Slice).

### Flow fractionator

Nuclei in samples from the flattened (right) V1 and processed using the flow fractionator were stained with DAPI and an antibody to NeuN and quantified by a flow cytometer (Collins et al., 2010; Young et al., 2012). The flow fractionator also requires the addition of a known number of Countbright Beads (Invitrogen) (Young et al., 2012) (Figure 1). Specifically, free-floating, DAPI-stained or DAPI+/NeuN+ double-stained nuclei from the main suspension samples were counted in tandem with a fixed volume of fluorescent Countbright beads (Invitrogen Inc.) using a Becton Dickson 5-laser LSRII flow cytometer (for details, see Collins et al., 2010; Young et al., 2012) (Figure 1). All samples were vigorously mixed before counts were determined, and all counts were made in duplicate to assess variability. The flow cytometry technician was blind to the sample attributes prepared by DJM and to the manual count data. All flow cytometry experiments were conducted in the Vanderbilt University Medical Center Flow Cytometry Shared Resource. Each of the 61 samples from right V1 was processed in duplicate using the flow fractionator method.

### Optical fractionator

This study also employed a traditional histological procedure to reveal cell bodies based upon the presence of staining for Nissl-substance (Nissl), as well as immunohistochemical staining for NeuN (Young et al., 2013), in which V1 is easily identifiable (Figure 1), for stereological quantification. Specifically, adjacent 1-in-10 series of sections cut from the left hemisphere on a freezing sledge microtome were processed for Nissl-substance using 1% thionin acetate (T3387 Sigma-Aldrich) dissolved to 0.025% into an acetate buffer (containing sodium acetate and acetic acid to a pH of 5.1–5.5) after mounting on glass slides and dried overnight in an oven set to 37°C, or free-floating with an antibody to NeuN. Sections were dehydrated in a graded series of alcohol (70, 95, 100%) followed by immersion in xylene prior to being coverslipped with Permount (SP15-500 Fisher). Neurons were apparent in both Nissl- and NeuN-stained materials because of the presence of a round nucleus. Glia were apparent in Nissl-stained sections because of their smaller size and darkly stained punctation. All stereological analyses were performed by a single investigator (DJM) using a computer-assisted stereology system consisting of a Nikon E80i light microscope with a motorized stage and the commercially available StereoInvestigator software (MicroBrightField Bioscience, Williston, VT). The volume of each region of interest was estimated using the Cavalieri principle (frame area = 500 × 500 μm) at 4× magnification (Table 1). An initial pilot study, in which sections were outlined at 2× magnification and counts were placed at 100× magnification using an oil immersion objective lens under Köhler illumination when the top of the nucleus, in the case of neurons, or the top of the cell profile, in the case of glia, came into focus within the probe dimensions (Schmitz and Hof, 2005), was performed to determine stereological parameters. Specifically, the pilot study indicated the appropriate number of sections to be investigated (*n* = 10), probe area (40 × 40 μm), probe height (10 μm), dual vertical guard zone height (2 μm), frame step (1966 × 2595 μm), frequency of thickness measurement (1 in 8; measured thickness: mean = 16.8 μm, s.e.m. = 0.31 μm) while maintaining an acceptably low coefficient of error [Gundersen (*m* = 1) CE < 0.025; 1st Schmitz and Hof CE < 0.024; 2nd Schmitz and Hof CE < 0.019] (Gundersen and Jensen, 1987; Schmitz and Hof, 2005).

In addition, these preliminary data indicated that the chromagens used to visualize cells or nuclei achieved full penetration of the tissue, based upon the homogeneity of cell counts across the depth of probes used to investigate NeuN-stained materials, and the failure to reject the null hypothesis, in Nissl-stained materials, that there was no difference in the number of cells allocated to sequential counting bins along the z-axis [*F*_(1,8)_ = 0.82, *P* > 0.39, *n* = 10 bins for a probe depth of 10 μm]. Additional analysis of the vertical (z) distribution of particles was performed at 100× magnification by recording the z-axis location of cells (nuclei; *n* = 1399) within the measured depth of tissue at sampling sites (*n* = 207) from across V1 in Nissl-stained material. A total of 640 sampling sites were investigated using the optical fractionator workflow to estimate cell and neuron number (mean number of sites per section = 32.4, 34.2; standard error of the mean (SEM) = 5.8, 4.8; NeuN, Nissl, respectively). The number of cells or neurons was calculated by multiplying the number of counts by the reciprocals of the area section fraction (asf), serial section fraction (ssf), and the tissue section fraction (tsf) (Schmitz and Hof, 2005). The percent of neuronal cells in brain slices investigated using stereology was calculated by taking the number of cells estimated using NeuN-stained sections divided by the number of cells estimated using Nissl-stained sections.

### Statistical analysis

The goals of this project were (1) to determine if manual and automated methods of counting single and double-stained nuclei in suspension volumes of homogenized tissue samples resulted in statistically indistinguishable estimates of total cell and neuron numbers from readily identifiable regions of interest in flattened cortical preparations, (2) to compare these results with estimates of the same parameters in the same region of interest from the other hemisphere in a single individual using stereological procedures on sectioned tissue, and (3) to determine the distribution of particles in the z-domain of Nissl-stained sections. Pursuant to the first goal, we determined manual counts as well as automated flow counts from at least two aliquots from each homogenized sample of flattened cortex used in this study (*n* = 61, Table 1). Next, we determined within-measures agreement by calculating the non-parametric T statistic on repeated measurements of each aliquot, testing the difference between means, such that a *P*-value smaller than 0.05 was taken to indicate a significant difference between the measurements (Table 2). We then used the Bland and Altman (1986) approach of comparing the differences between the measurements to the means of the measurements in our analysis of the isotropic fractionator and the flow fractionator data (Figures S1–S4). In addition, we report Lin's concordance correlation, a statistic that approximates the variation of the data about the linear regression (Lin, 1989) (Table 2). Pursuant to the second goal of comparing results from samples of flattened cortex with results from coronal brain slices, we calculated the density of cells or neurons per cubic millimeter by taking the parameter estimate of the sample divided by the measured volume of that sample. For example, when using the optical fractionator, the investigator draws an outline of the region of interest in each of the 10 sections selected for analysis. The Cavalieri method then utilizes this outline to estimate the surface area of that section. The volume of the section is then calculated by taking the surface area multiplied by the thickness of the section as it was originally cut on the freezing microtome. The density of cells or neurons is then calculated by dividing the number of cells or neurons estimated using the optical fractionator by the volume of each section determined by the Cavalieri method. The 10 sections are then ordered by density. In contrast, the surface area of each flattened cortical sample was measured using ImageJ and multiplied by the depth of V1 cortex measured in the intact left hemisphere cut as brain slices to determine the volume of each sample. The average of the isotropic fractionator and flow fractionator estimates was then divided by the volume of each sample to calculate cell or neuronal density (Table 1). In addition, these averages were ordered by density and placed into 10 bins of approximately six samples each (the 10th bin contained seven samples). The non-parametric T statistic was then calculated on the ordered (a) flattened cortical sample and (b) intact brain section density estimates (Table 2). Pursuant to the third aim of determining the distribution of particles in the z-domain of brain slices from tissue fixed before sectioning while frozen on a sliding microtome, we normalized the z-axis location of each cell within the corresponding depth of the sampling site in which it was observed and divided this normalized data into 10 bins corresponding to percentiles of tissue depth. We then used linear regression to investigate the presence of a statistically distinguishable relationship between the frequency and location of cell counts along the z-axis.

**Table 2:** **Summary statistics table**.

**Analysis**	**Tissue**	**Quantification**	**Parameter**	**Test statistic**	***P*-value**	**Sample**	**Figures**
Within	Flat	Flow cytometry	Total cells	Nonparametric paired *T*-test	0.486	*n* = 61	Figure S1
				Lin's concordance statistic	0.877	*n* = 61	
	Flat	Neubauer chamber	Total cells	Nonparametric paired *T*-test	0.221	*n* = 61	Figure S2
	Slice	Stereology	Total cells	Gundersen CE (*m* = 1)	0.027	*n* = 324	
			Total neurons	Gundersen CE (*m* = 1)	0.018	*n* = 316	
			Volume (Nissl)	Gundersen CE (*m* = 1)	0.017	*n* = 324	
			Volume (NeuN)	Gundersen CE (*m* = 1)	0.008	*n* = 316	
Between	Flat vs. Flat	Flow cytometry vs. Neubauer chamber	Total cells	Nonparametric paired *T*-test	0.239	*n* = 61	Figure S3
				Lin's concordance statistic	0.892	*n* = 61	
	Flat vs. Flat	Flow cytometry vs. Neubauer chamber	Percent neuron	Nonparametric paired *T*-test	0.124	*n* = 61	Figure S4
				Lin's concordance statistic	0.416	*n* = 61	
	Slice vs. Flat	Stereology vs. Isotropic fractionator	Cell density	Nonparametric paired *T*-test	0.922	*n* = 10	Figure 2
				Lin's concordance statistic	0.787	*n* = 10	
			Neuron density	Nonparametric paired *T*-test	0.027	*n* = 10	Figure 2
				Lin's concordance statistic	0.599	*n* = 10	

Summary table showing the statistical tests performed to assess variation in measurements taken with a single quantification method (Within) and between quantification methods (Between) in tissue processed as flattened cortical samples (Flat) or brain slices (Slice). P-values lower than 0.05 were considered significant. See Methods for full details of statistical analysis.

## Results

Our investigation of chimpanzee (*Pan troglodytes*) primary visual cortex (V1, Figure 1) revealed excellent agreement between the estimated number of cells or neurons derived from tissue processed as samples from flattened cortex and quantified manually using the isotropic fractionator (Herculano-Houzel and Lent, 2005) and automatically using the flow fractionator (Collins et al., 2010; Young et al., 2012), or from tissue processed as coronal sections and quantified using the computer-assisted optical fractionator method (Schmitz and Hof, 2005, Figure 2). Specifically, the stereological estimate (961 million) of total cell number from coronal slices of V1 was 94.6% of the estimate based upon manual and automated counts (1 billion) of flattened cortex. The percent of all the cells that are neurons, calculated from flattened samples (65.4%), was 90.3% of the percent estimated using stereology (72.4%). Manual and automated count estimates (658 million) of the number of neurons in V1 were 95.6% of the stereological estimate (695 million, Table 1).

**Figure 2:**
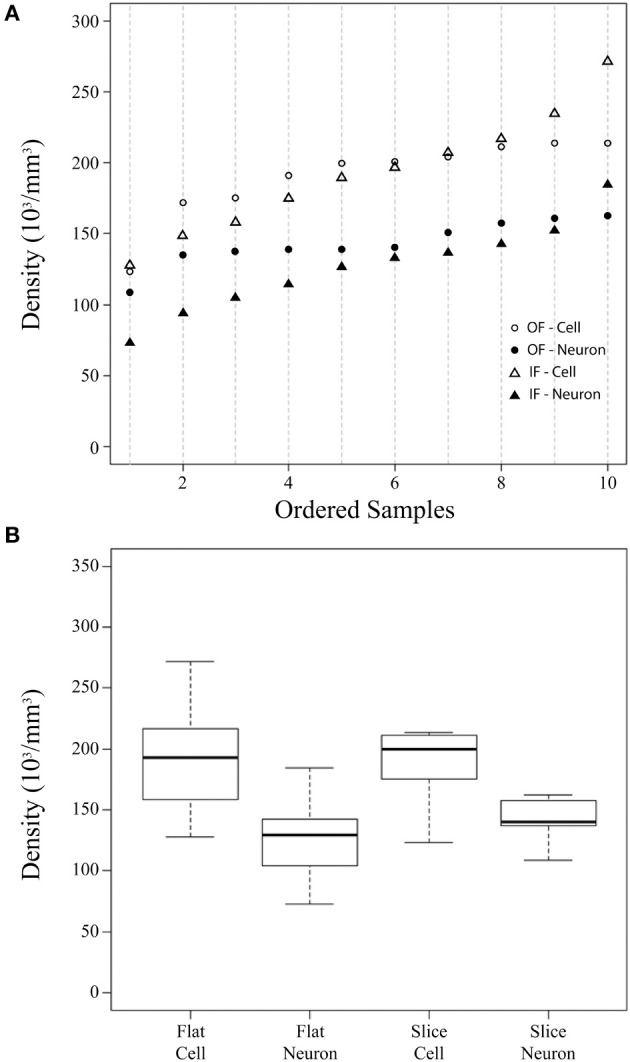
**Summary cellular density plots**. Summary plots showing the density (in thousands of cells per mm^3^) of cells and neurons estimated from flattened cortical samples (Flat) and brain slices (Slice) **(A,B)**. In these plots, the flattened cortical sample estimate is the average of counts using the Neubauer chamber and flow cytometry for each sample. Estimates from flattened cortical samples were ordered and placed into 10 bins of 6 sequential values (the 10th bin contains 7 values, *n* = 61) and are shown for all cells by an open triangle and for neurons by a closed triangle **(A)**. Stereological density estimates from brain slices (Slice) were ordered (*n* = 10) and are shown for all cells by open circles and for neurons by closed circles **(A)**. **(B)** Is a boxplot depicting the median (thick line), inter-quartile range (box), minimum (lower dotted error bar), and maximum (upper dotted error bar) cellular densities in flattened cortical samples and brain slices.

The current investigation also found that V1 volume of the right hemisphere estimated from images of flattened samples using ImageJ (5.3 cm^3^) was within 10% of V1 volume in the left hemisphere estimated from serial sections using the Cavalieri stereological method (4.8 cm^3^, Table 1). Thus, the density of cells in flattened samples of V1 ranged from 110,783 to 329,632 cells/mm^3^, with an average of 193,867 cells/mm^3^ (Figure 2, Table 1). The density of neurons in flattened samples of V1 ranged from 52,936 to 234,039 neurons/mm^3^, with an average of 126,823 neurons/mm^3^. The density of cells in coronal slices of V1 ranged from 123,263 to 213,806 cells/mm^3^, with an average of 197,331 cells/mm^3^. The density of neurons in coronal slices of V1 ranged from 108,656 to 162,511 neurons/mm^3^, with an average of 146,008 neurons/mm^3^. These results appear in Figure 2 and Table 1. Therefore, our investigation revealed that the density of cells and neurons from flattened samples were 98.2 and 86.9% of the stereological estimates, respectively.

Corroborating the results of previous studies (Collins et al., 2010; Young et al., 2012), we observed excellent agreement between repeated measurements using the isotropic fractionator (non-parametric *T*-test, *P* = 0.221, Lin's Concordance = 0.812, *n* = 61) and the flow fractionator (non-parametric *T*-test, *P* = 0.486, Lin's Concordance = 0.877, *n* = 61) to estimate the total number of cells. Similarly, we observed low within-sample variance when using the optical fractionator to estimate the number of neurons [Gundersen coefficient of error (CE) = 0.018 (Gundersen and Jensen, 1987); 1st CE = 0.028, and 2nd CE = 0.021 (Schmitz and Hof, 2005)] and the total number of cells [Gundersen CE = 0.027 (Gundersen and Jensen, 1987); 1st CE = 0.023, and 2nd CE = 0.018 (Schmitz and Hof, 2005)], or when using the Cavalieri method to estimate V1 volume (Gundersen CE = 0.012, Gundersen and Jensen, 1987). In addition, we observed no difference when comparing estimates of the number of neurons (non-parametric *T*-test, *P* = 0.124, *n* = 10) or the total number of cells (non-parametric *T*-test, *P* = 0.239, *n* = 61) based on manual counts under the microscope and automated counts using the flow cytometer (Table 1).

Our investigation employed a constant estimate of the cortical depth of V1 (~1.8 mm, *n* = 42) to determine the volume of samples from flattened cortex because estimates based upon sample weights were more variable, likely as a result of the inclusion of some white matter. As noted, this approach produced a volume estimate of right V1 that was 10% larger (5.3 mm^3^) than the (4.8 mm^3^) estimate obtained using the Cavalieri method in brain slices from left V1. Despite this difference, our calculations of total cellular density in chimpanzee V1 comparing an average of the isotropic fractionator and the flow fractionator with the optical fractionator were statistically indistinguishable (non-parametric *T*-test, *P* = 0.922, *n* = 10). In contrast, our calculations of neuronal density using these techniques were statistically distinguishable (non-parametric *T*-test, *P* = 0.027, *n* = 10, Table 2). The increased estimate of V1 volume from flattened cortex highlights the need for the anatomical specificity available in stained brain slices and may account for the discrepancy between average neuronal density estimates using these techniques (see Discussion).

**Figure 3:**
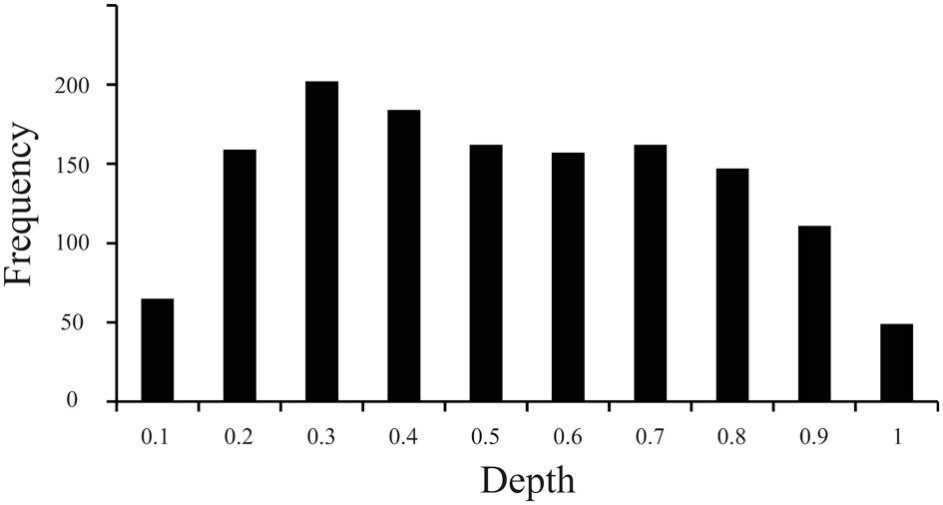
**Distribution of particles along the z-axis of Nissl-stained brain slices**. Histogram of data based on 1399 cells (nuclei) from 207 sampling sites throughout V1 illustrating the frequency of cell counts (Y axis) normalized over the depth (X axis) of sections fixed before cutting frozen on a sliding microtome and mounted before staining for Nissl substance. The convention here is that bins to the left (e.g., bin #0.1) are near the coverslip or the exposed edge of tissue during staining (superficial), and bins to the right (e.g., bin #1) are near the glass slide (deep).

The current work revealed that the distribution of particles along the z-domain of sectioned materials fixed before sectioning while frozen on a sliding microtome and mounted on a glass slide before staining for Nissl-substance was vulnerable to the “lost caps” phenomena (Baryshnikova et al., 2006), and exhibited a linear decrease in cell density with increasing tissue depth (superficial = near coverslip, deep = near glass slide, Figure 3). Specifically, our measurements indicated that the top and bottom 10% of tissue (bins 0.1 and 1, respectively) each exhibited ~40% of the average z-domain cell count (“lost caps,” see Baryshnikova et al., 2006). Including the entire z-axis in the stereological probe would therefore have resulted in an underestimate of the cellular population by ~12.9%. After excluding the top and bottom 10% of tissue, we identified a linear decrease in the density of cell counts as the depth of tissue increased, such that tissue near the slide (deep) contained ~20% fewer counts than (superficial) bins near the coverslip [*F*_(1,6)_ = 7.7, *P* = 0.032, Adjusted *R*^2^ = 0.48]. The current work, based upon the results of a pilot study indicating the use of 2 μm guard zones, effectively sampled the tissue from 2 to 12 μm of the average 16.8 μm available, or from the 12th to 71st percentiles along the z-axis. Based on the average counts per bin after excluding the top and bottom 10 percentiles (bin #0.1 [adjacent to the coverslip], and bin #1 [adjacent to the glass slide]), our calculations indicate that this sampling range (12–71%, or bins 2–7) resulted in an estimate of cell number approximately 2.6% above the sample mean.

## Discussion

Our estimates of cellular and neuronal populations in the primary visual cortex of a chimpanzee using the isotropic fractionator, the flow fractionator or the optical fractionator agree within 5% (Table 1). This work also contributes data indicating that the measurement of V1 volume from flattened preparations agrees with stereological estimates from brain slices within 10% (Table 1). Our investigation therefore provides evidence of the agreement between estimates of the density of cells and neurons based upon sampling flattened cortex or brain slices (Figure 2).

We provide data on the volume of chimpanzee V1 (4.8–5.3 cm^3^) consistent with the 4.64 cm^3^ (De Sousa et al., 2010) and 5.52 cm^3^ (Bush and Allman, 2004) estimates from previous studies. In particular, our stereological estimate of 4.8 cm^3^ supports the De Sousa et al. (2010) study that also used the Cavalieri method to estimate the volume of V1 in the left hemisphere of chimpanzees at 4.64 cm^3^. The difference between our estimates in the left (4.8 cm^3^) and right hemispheres (5.3 cm^3^) may reflect inter-hemispheric asymmetry in V1 volume, given that previous work has reported differences in humans of 5% (Murphy, 1985) and 13% (Andrews et al., 1997). This difference in V1 volume may be related to behavioral specializations as has been observed in the primary motor cortex related to hand preference (Sherwood et al., 2007a,b; Hopkins et al., 2010). On the other hand, it is possible that mechanical deformation of the cortical sheet induced during the flattening procedure increased our surface area measurement which, when combined with depth measurements from the intact left hemisphere, produced a slight over-estimate of V1 volume in the right hemisphere. However, given that our estimates were within 10% of each other, and that our calculations fall well within the range suggested by previous studies and exhibit inter-hemispheric asymmetry similar to what has been reported in humans, these results suggest that our measurements provide reasonable estimates of the volume of V1 in the chimpanzee brain.

Results from our investigation of the distribution of particles along the z-axis in sectioned materials stained for Nissl after mounting suggest that the use of guard zones to exclude the top and bottom 10% of tissue avoids underestimating the total number of cells by approximately 13% (effect of “lost caps,” Figure 3, see Baryshnikova et al., 2006). However, the implementation of 2 μm guard zones resulted in an effective probe sampling range of the 12–71st percentiles along the z-axis which resulted in a slight overestimation of cell number by approximately 2–3%, based upon the observed decrease in cell density as the observer approaches the glass slide. However, previous work has reported an increase in cell density as the observer moves from the tissue near the coverslip toward the glass slide upon which the tissue was mounted, and the absence an effect of “lost caps,” such that cells near sectioned edges were not notably fewer in number than in the middle of the brain slice (Carlo and Stevens, 2011). The inverse effects observed in these two studies are likely explained by the observation that the tissue Carlo and Stevens (2011) measured was processed in a manner identical to that employed in the current investigation, except for two things: (1) Carlo and Stevens (2011) report flipping the tissue such that the edge of the tissue immediately adjacent to but above the knife blade during sectioning was positioned closest to the coverslip in the final stained product, whereas the current study did not flip the tissue in this manner, and (2) Carlo and Stevens (2011) measured brain tissue that underwent perfusion fixation prior to sectioning, while the current investigation measured tissue that underwent a saline flush during perfusion and was subsequently fixed by immersion before sectioning. The opposing gradients of cell loss are therefore likely the result of the same phenomena in both studies such that cells immediately above the microtome blade during sectioning exhibit an increased density, an effect of tissue compression that occurs prior to mounting the tissue (Baryshnikova et al., 2006), and are simply being measured across the tissue in opposing directions. Similarly, the presence of “lost caps” in the current investigation but the absence of this effect in Carlo and Stevens (2011) data is therefore likely the result of whether the brain was fixed by perfusion (resulting in no lost caps), or not (resulting in approximately 60% cell loss at the cut edges). This conclusion is supported by similar observations from different labs that cells are lost at cut edges in immersion fixed (Andersen and Gundersen, 1999; Bahney and von Bartheld, 2014) but not perfusion fixed tissues (Gardella et al., 2003). Thus, these studies together suggest that compression occurs regardless of fixation and is characterized by an effect somewhere between the currently reported 20% (clearance angle = 19°) and previously reported 50% (clearance angle = 7.5°, Carlo and Stevens, 2011) decrease in cell density as the observer moves away from the edge of tissue adjacent to and above the knife edge during sectioning. In addition, perfusion fixation appears to mitigate the effect of “lost caps,” which results in the loss of a significant number of cells or particles near the surface of sections from immersion-fixed tissues (~60%). Given that the ultimate goal of stereological investigations is to estimate biological parameters of interest in such a way that the original relationships among those parameters are not systematically distorted, our data suggest that the use of guard zones in these tissue preparations are critical, and that awareness of the extent to which the stereological probe employed samples a known distribution of objects within the available tissue can provide reasonable estimates, for example, of the number of cells and neurons. In summary, our data provide evidence in support of the traditional stereological approach to focus on sampling the “core” of sectioned material, because this avoids the largest source of bias due to the “lost caps” effect at tissue edges (von Bartheld, 2012). In addition, our data provide evidence to suggest that the use of guard zones resulting in an effective sampling range from anywhere within the 10–30th to the 70–90th percentiles produce results within 3–4% of the observed or sample mean.

This study corroborated previous work (Young et al., 2012) comparing the total number of cells in a sample measured using the isotropic fractionator and the flow fractionator (Figures S1–S3). We found that the total number of cells estimated from flattened cortical samples was 5% greater than the stereological estimate (Table 1). However, our stereological investigation likely overestimated the actual number of cells in our sample by approximately 3%, resulting in a discrepancy between hemispheres of approximately 8%. Given that previous investigations of human V1, as well as the current volumetric analysis, indicate inter-hemispheric differences on the order of 5–13% (Murphy, 1985; Andrews et al., 1997), these data suggest that the observed differences in cell number reflect biological asymmetry between the hemispheres. The close agreement between these results and previous studies therefore strongly suggests that each of these distinct techniques estimate a similar parameter, the total number of cells in chimpanzee V1.

This work provides evidence in agreement with previous studies investigating the percentage of neuronal cells in samples measured using the isotropic fractionator and the flow fractionator (Collins et al., 2010; or Figure S4). It should be noted that the estimate of the absolute number of neurons in tissue samples from flattened cortex is a derivative of the estimate of the total number of cells using DAPI. This contrasts with the stereological estimate of neuron number, which is based upon an independent run of the optical fractionator in a series of sections stained for NeuN, adjacent to the Nissl-stained series. The percentage of neuronal cells based upon our stereological investigation is calculated as the number of cells in NeuN-stained sections divided by the number of cells in Nissl-stained sections. Therefore, our comparison of the number of neurons using these techniques is two-fold: (a) we compared the percentage of all the neuronal cells, and (b) we compared the absolute number of neurons estimated. We found that the mean percentage of neuronal cells in chimpanzee V1 estimated from flattened cortical samples was 65.4%, while the mean percentage of neuronal cells estimated using stereology was 72.4%. Given that our stereological analysis likely produced a ~3% overestimate of cell number, the percentage of neuronal cells estimated from brain slices may be approximately ~74%. This difference in the percentage of neuronal cells is likely due to the inclusion of a minimal amount of white matter in the flattened cortical sample preparation. Next, we calculated the absolute number of neurons and discovered that the estimate from flattened cortical samples (658 million) was 5% lower than the stereological estimate of 695 million (Table 1). Given that previous evidence indicates a >5% rightward bias in V1 size and cell number, the observation that right V1 contains 5% fewer neurons suggests that neurons may be lost during implementation of the isotropic fractionator method (Clarke and Oppenheim, 1995). Although future research may further clarify this issue, the reasonably close (~10%) agreement between these estimates suggest that our quantification procedures approximate the same parameter, the total number of neurons in chimpanzee V1.

Our data provide evidence that there are approximately 195,000 (193,867–197,331) cells per mm^3^ of chimpanzee V1, out of which around 136,000 (126,823–146,008) are neurons. These data differ somewhat from the results of a previous study of cellular density in chimpanzee V1 which reported ~330,000 cells per mm^3^, out of which 208,930 were neurons (Lewitus et al., 2012). Interestingly, both of these results place the density of neurons in chimpanzee V1 in range of the upper 60 percentiles. However, the discrepancy between the absolute density of neuronal and non-neuronal cells in these studies is likely due to a combination of factors including tissue preparation, the type of fixation and the histological staining protocol employed. For instance, Lewitus et al. (2012) excluded layer 1 from their estimates as a result of variable tissue quality, and measured cortical thickness only twice for each section investigated, the combination of which may have contributed to the higher cellular densities they observed. However, our results are in agreement with this and other published studies indicating a high density of neurons in primary visual cortex, particularly in primates (Collins et al., 2010, 2013; Campi et al., 2011; Lewitus et al., 2012).

The density of cells and neurons based on flattened samples were 98.2 and 86.9% of the stereological estimates, respectively. However, the observation that our stereological data probably overestimated (~3%) the total number of cells may account for the observed ~2% difference in cell density between hemispheres. These results demonstrate that the density of cells can be determined with high precision, whether measured using traditional stereological tools or with more recently developed techniques such as the isotropic fractionator and flow fractionator. In our hands, these distinct methodologies produced estimates of the density of neurons that agreed within 15%. The source of this discrepancy may involve the inclusion of a minimal amount of white matter in flattened cortical samples during dissection, the slight overestimation of regional volume because of deformations in the cortical sheet induced during the flattening procedure, the loss of neuronal cells, or some combination of these. Despite these relatively small differences, our ability to replicate the composition and density of cellular populations in the chimpanzee primary visual cortex provides evidence supporting our ability to compare results from distinct methodologies in determining the distribution of different types of cells in the brain.

## Conclusions and significance

Our research indicates the reproducibility of estimates of the cellular composition of brain tissue using distinct tissue processing and quantification methodologies when the accurate identification of a single cortical region of interest is possible. Our results thus demonstrate the validity of using the isotropic fractionator, flow fractionator, or optical fractionator to obtain cell and neuron counts. Ultimately, these results facilitate a more comprehensive understanding of neurobiological circuitry by presenting investigators with evidence that these techniques estimate the same parameters of interest, allowing the investigator an additional line of evidence to aid in the interpretation of experimental results.

Our results are also significant because they indicate that V1 in chimpanzees, our closest living genetic relatives, conforms to the general finding in primates that V1 contains more densely packed neurons than other cortical areas (Rockel et al., 1980; Carlo and Stevens, 2013; Collins et al., 2013; Young et al., 2013). This means that, beyond the observation that layer 4 contains small neurons, each of the cortical layers in V1 contains neurons that are on average smaller than in other cortical areas. Smaller neurons tend to have fewer synaptic inputs (Jacobs et al., 2001; Elston et al., 2005) and are thus thought to be less likely to dramatically transform incoming sensory signals. Primate brains are known to contain a large proportion of neocortical tissue devoted to visual perception. Furthermore, compared to other mammals, V1 in primates has the specialized task of distributing sensory input to cortical regions throughout the visual system that are integral for visual percepts. Accordingly, our findings are consistent with the observation that primate V1 is characterized by small, densely packed neurons, which may have facilitated the evolution of primate V1 into a region particularly effective at providing multiple downstream cortical regions with the relatively raw sensory input necessary for higher-order functional operations.

## Author contributions

Daniel J. Miller and Jon H. Kaas contributed to the conception and design of the work. Daniel J. Miller, Pooja Balaram, and Nicole A. Young made substantial contributions to the acquisition and analysis of the data. Daniel J. Miller, Pooja Balaram, Nicole A. Young, and Jon H. Kaas all participated in the interpretation of the data. Daniel J. Miller drafted the manuscript and Pooja Balaram, Nicole A. Young, and Jon H. Kaas revised the manuscript for critically important intellectual content. Daniel J. Miller, Pooja Balaram, Nicole A. Young, and Jon H. Kaas each had access to all aspects of this work, take responsibility for the accuracy and integrity of this work in its entirety and gave final approval of the version to be published.

### Conflict of interest statement

The authors declare that the research was conducted in the absence of any commercial or financial relationships that could be construed as a potential conflict of interest.

## Supplementary material

Figure S1 **Analysis of flow cytometry estimates**. Repeated measures graph depicting the agreement between the first (red) and second (blue) density measurements on ordered samples **(A)**. The vertical dotted lines indicate every 5th sample **(A)**. Bland-Altman plot showing the agreement between the first and second measurement taken from each sample by comparing the difference between repeated measurements on the Y axis against the mean of each sample estimate plotted along the X axis **(B)**. The blue dotted line indicates the sample mean, the green dotted lines indicate the 95% interval, and the red dotted line indicates a single standard deviation from the mean **(B)**. All density estimates are in thousands of nuclei per mm^3^ **(A,B)**.

Figure S2 **Analysis of manual counts using the Neubauer chamber**. Repeated measures graph depicting the agreement between the first (red) and second (blue) density measurements on ordered samples **(A)**. The vertical dotted lines indicate every 5th sample **(A)**. Bland-Altman plot showing the agreement between the first and second measurement taken from each sample by comparing the difference between repeated measurements on the Y axis against the mean of each sample estimate plotted along the X axis **(B)**. The blue dotted line indicates the sample mean, the green dotted lines indicate the 95% interval, and the red dotted line indicates a single standard deviation from the mean **(B)**. All density estimates are in thousands of nuclei per mm^3^ **(A,B)**.

Figure S3 **Analysis of flattened cortical sample estimates**. Repeated measures graph depicting the agreement between the average density count using the Neubauer chamber (red) and the flow cytometer (blue) on ordered samples **(A)**. The vertical dotted lines indicate every 5th sample **(A)**. Bland-Altman plot showing the agreement between these quantification procedures by comparing the difference between estimates on the Y axis against the mean of each sample estimate plotted along the X axis **(B)**. The blue dotted line indicates the sample mean, the green dotted lines indicate the 95% interval, and the red dotted line indicates a single standard deviation from the mean **(B)**. All density estimates are in thousands of nuclei per mm^3^ **(A,B)**.

Figure S4 **Analysis of neuronal percentage**. Repeated measures graph depicting the agreement between the average neuronal percentage using the Neubauer chamber (red) and the flow cytometer (blue) on ordered samples **(A)**. The vertical dotted lines indicate individual samples **(A)**. Bland-Altman plot showing the agreement between these quantification procedures by comparing the difference between estimates on the Y axis against the mean of each sample estimate plotted along the X axis **(B)**. The blue dotted line indicates the sample mean, the green dotted lines indicate the 95% interval, and the red dotted line indicates a single standard deviation from the mean **(B)**. All density estimates are in thousands of nuclei per mm^3^ **(A,B)**.
