# Supplementary figures and images for: Three counting methods agree on cell and neuron number in chimpanzee primary visual cortex
Source: Front Neuroanat. 2014 May 16;8:36. doi: 10.3389/fnana.2014.00036 (PMC4032965; doi:10.3389/fnana.2014.00036)

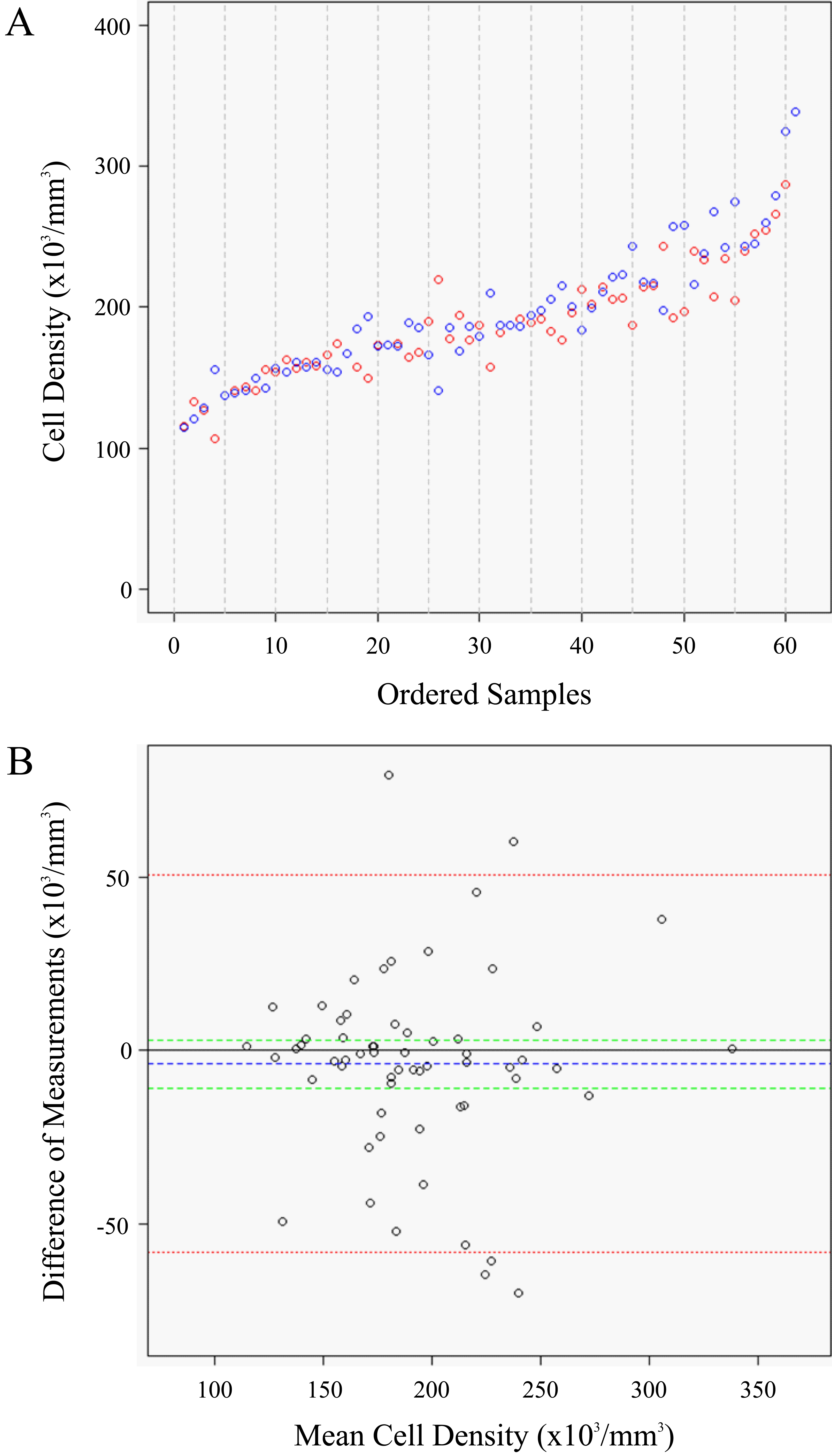

Supplement: Figure S1 — Analysis of flow cytometry estimates. Repeated measures graph depicting the agreement between the first (red) and second (blue) density measurements on ordered samples (A). The vertical dotted lines indicate every 5th sample (A). Bland-Altman plot showing the agreement between the first and second measurement taken from each sample by comparing the difference between repeated measurements on the Y axis against the mean of each sample estimate plotted along the X axis (B). The blue dotted line indicates the sample mean, the green dotted lines indicate the 95% interval, and the red dotted line indicates a single standard deviation from the mean (B). All density estimates are in thousands of nuclei per mm3 (A,B). [file Presentation1.ZIP › 88556_Miller_Suppl_Figure_2.TIF]

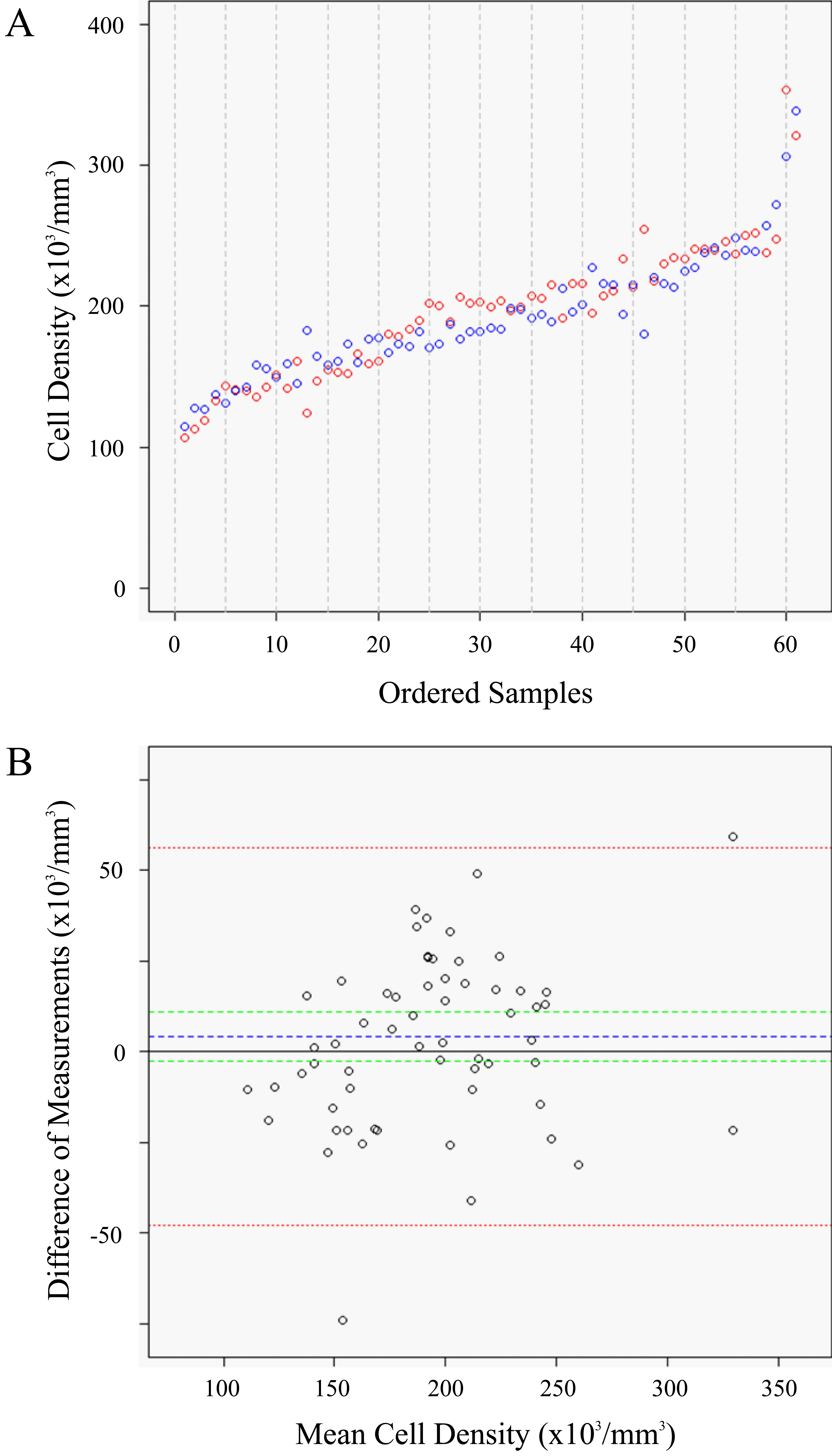

Supplement: Figure S1 — Analysis of flow cytometry estimates. Repeated measures graph depicting the agreement between the first (red) and second (blue) density measurements on ordered samples (A). The vertical dotted lines indicate every 5th sample (A). Bland-Altman plot showing the agreement between the first and second measurement taken from each sample by comparing the difference between repeated measurements on the Y axis against the mean of each sample estimate plotted along the X axis (B). The blue dotted line indicates the sample mean, the green dotted lines indicate the 95% interval, and the red dotted line indicates a single standard deviation from the mean (B). All density estimates are in thousands of nuclei per mm3 (A,B). [file Presentation1.ZIP › 88556_Miller_Suppl_Figure_3.TIF]

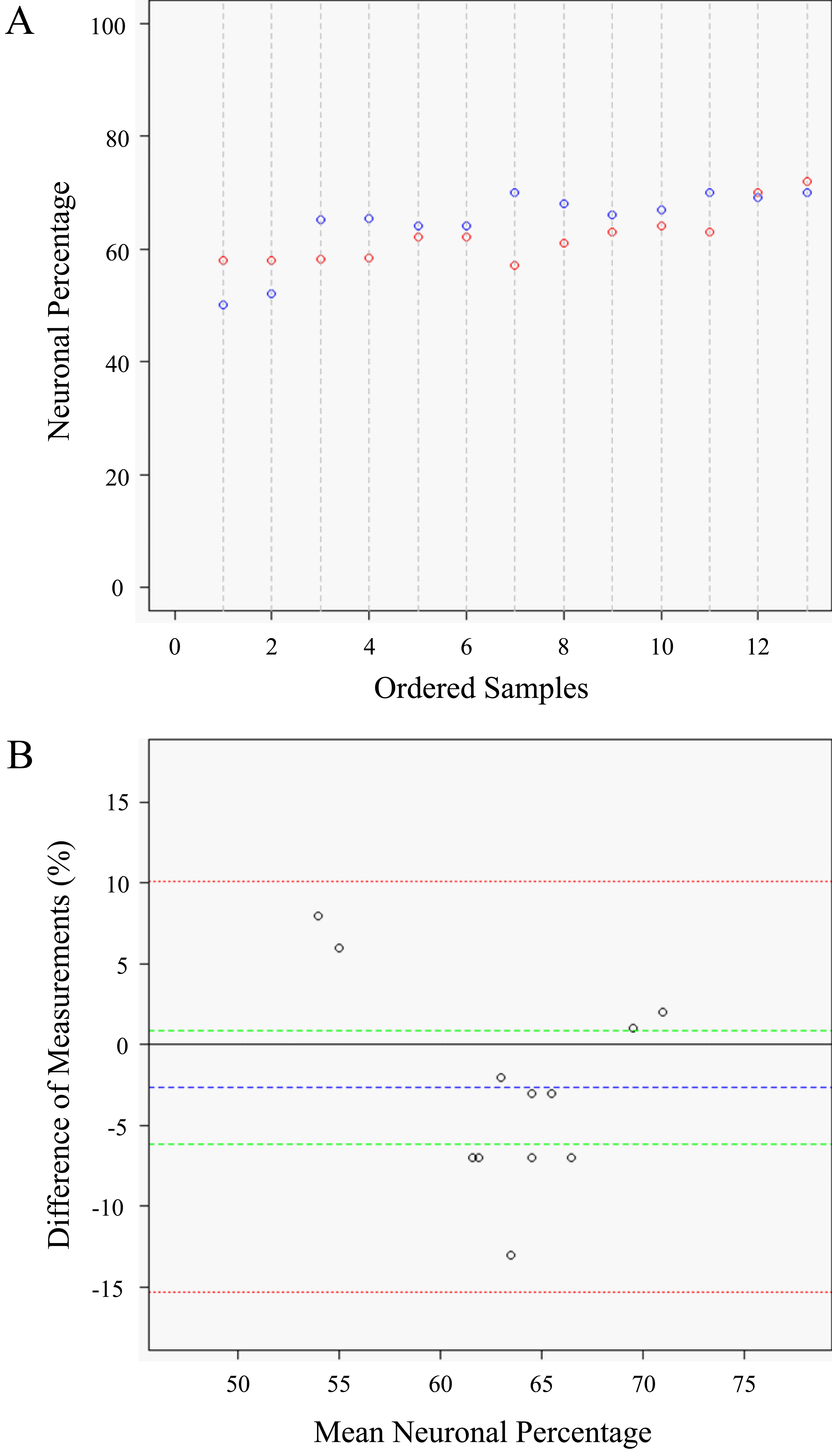

Supplement: Figure S1 — Analysis of flow cytometry estimates. Repeated measures graph depicting the agreement between the first (red) and second (blue) density measurements on ordered samples (A). The vertical dotted lines indicate every 5th sample (A). Bland-Altman plot showing the agreement between the first and second measurement taken from each sample by comparing the difference between repeated measurements on the Y axis against the mean of each sample estimate plotted along the X axis (B). The blue dotted line indicates the sample mean, the green dotted lines indicate the 95% interval, and the red dotted line indicates a single standard deviation from the mean (B). All density estimates are in thousands of nuclei per mm3 (A,B). [file Presentation1.ZIP › 88556_Miller__Suppl_Figure_4.TIF]

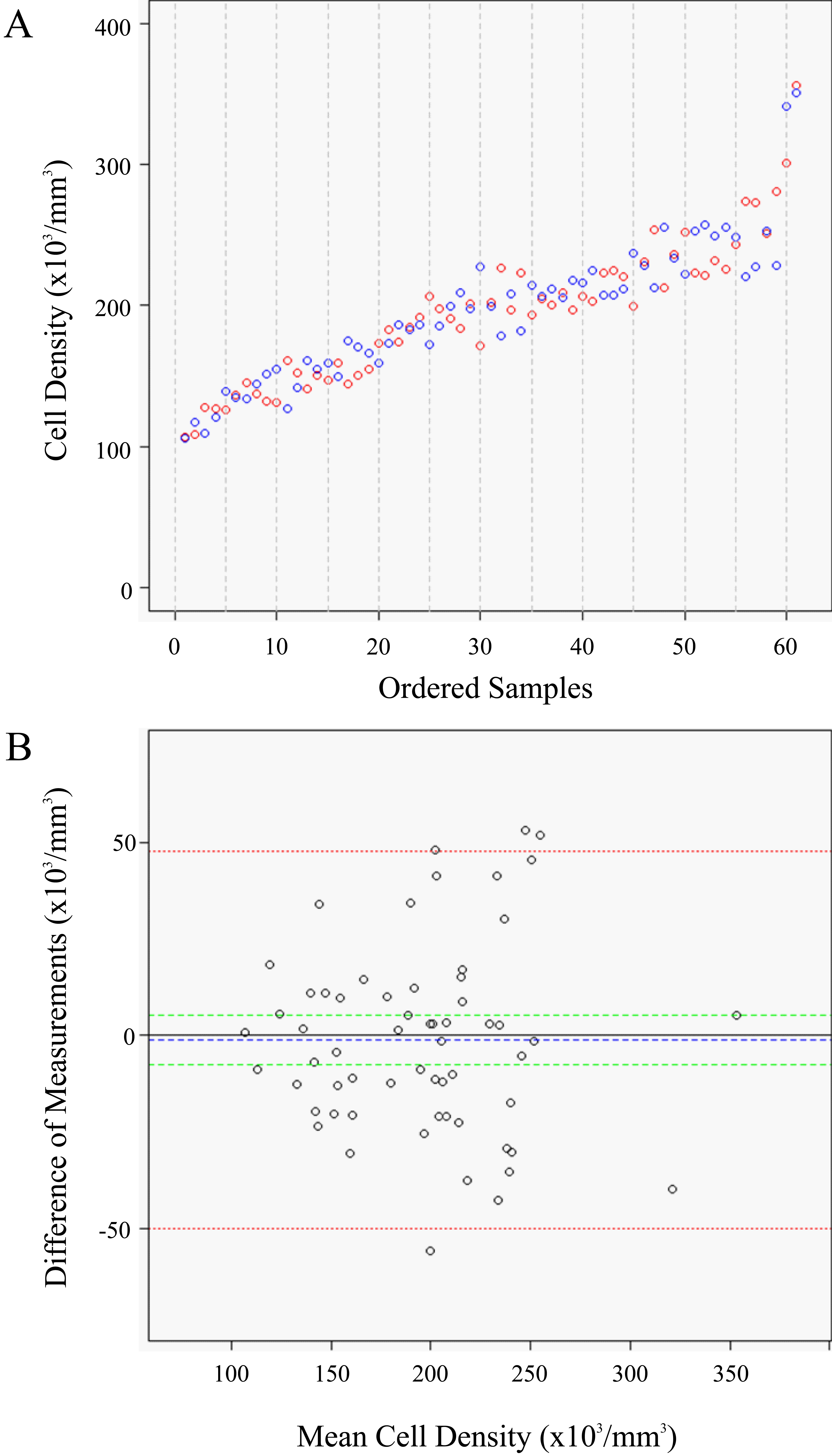

Supplement: Figure S1 — Analysis of flow cytometry estimates. Repeated measures graph depicting the agreement between the first (red) and second (blue) density measurements on ordered samples (A). The vertical dotted lines indicate every 5th sample (A). Bland-Altman plot showing the agreement between the first and second measurement taken from each sample by comparing the difference between repeated measurements on the Y axis against the mean of each sample estimate plotted along the X axis (B). The blue dotted line indicates the sample mean, the green dotted lines indicate the 95% interval, and the red dotted line indicates a single standard deviation from the mean (B). All density estimates are in thousands of nuclei per mm3 (A,B). [file Presentation1.ZIP › 88556_Miller_Suppl_Figure_1.TIF]
